# Alcohol Use Disorder, Neurodegeneration, Alzheimer’s and Parkinson’s Disease: Interplay Between Oxidative Stress, Neuroimmune Response and Excitotoxicity

**DOI:** 10.3389/fncel.2020.00282

**Published:** 2020-08-31

**Authors:** Haziq Kamal, Geok Chin Tan, Siti Fatimah Ibrahim, Mohd. Farooq Shaikh, Isa Naina Mohamed, Rashidi M. Pakri Mohamed, Adila A. Hamid, Azizah Ugusman, Jaya Kumar

**Affiliations:** ^1^Department of Physiology, Faculty of Medicine, Universiti Kebangsaan Malaysia, Kuala Lumpur, Malaysia; ^2^Department of Pathology, Faculty of Medicine, Universiti Kebangsaan Malaysia, Kuala Lumpur, Malaysia; ^3^Neuropharmacology Research Laboratory, Jeffrey Cheah School of Medicine and Health Sciences, Monash University Malaysia, Selangor, Malaysia; ^4^Department of Pharmacology, Faculty of Medicine, Universiti Kebangsaan Malaysia, Kuala Lumpur, Malaysia; ^5^Department of Family Medicine, Faculty of Medicine, Universiti Kebangsaan Malaysia, Kuala Lumpur, Malaysia

**Keywords:** alcohol, neurodegeneration, excitotoxicity, neuroinflammation, neuroimmune, Alzheimer, Parkinson, oxidative

## Abstract

Alcohol use disorder (AUD) has been associated with neurodegenerative diseases such as Alzheimer’s and Parkinson’s disease. Prolonged excessive alcohol intake contributes to increased production of reactive oxygen species that triggers neuroimmune response and cellular apoptosis and necrosis via lipid peroxidation, mitochondrial, protein or DNA damage. Long term binge alcohol consumption also upregulates glutamate receptors, glucocorticoids and reduces reuptake of glutamate in the central nervous system, resulting in glutamate excitotoxicity, and eventually mitochondrial injury and cell death. In this review, we delineate the following principles in alcohol-induced neurodegeneration: (1) alcohol-induced oxidative stress, (2) neuroimmune response toward increased oxidants and lipopolysaccharide, (3) glutamate excitotoxicity and cell injury, and (4) interplay between oxidative stress, neuroimmune response and excitotoxicity leading to neurodegeneration and (5) potential chronic alcohol intake-induced development of neurodegenerative diseases, including Alzheimer’s and Parkinson’s disease.

## Introduction

According to the World Health Organization, 5.3% of all global deaths and 5.1% of the global burden of disease and injury are attributable to alcohol use disorder (AUD). Alcohol abuse increases the risks of both communicable and non-communicable diseases (World Health and Organization, 2019). Sudden cessation from heavy drinking results in manifestations of various physical symptoms, known as Alcohol Withdrawal Syndrome (AWS), and are primarily evident during the first 6–96 h following the last alcohol intake. AWS is often characterized by autonomic hyperactivity, agitation, hallucination and seizures (Mirijello et al., 2015; Jesse et al., 2017). Over the years, the majority of the alcohol-related studies focused on positive and negative reinforcement effects of the beverage (Salling et al., 2016; Mohamed et al., 2018a,b).

Alcohol-dependent individuals are prone to develop neurocognitive impairment and even neurodegenerative disorders (De la Monte and Kril, 2014). Findings from numerous investigations showed increased risk of neurodegenerative disorders such as dementia, and Parkinson’s Disease with excessive alcohol consumption (Eriksson et al., 2013; Lafortune et al., 2014; Zhang D. et al., 2014; Heymann et al., 2016; Schwarzinger et al., 2018). In this review, we described alcohol-induced oxidative stress, heightened glutamatergic excitotoxity, exacerbated neuroimmune response, and their collective effects leading to neurodegeneration and potential association with certain neurodegenerative diseases, such as Alzheimer’s disease and Parkinson’s disease.

## AUD-Induced Neurodegeneration

Chronic excessive alcohol intake often leads to cognitive impairment with white matter (WM) atrophy, axonal loss and demyelination at brain regions, including the hippocampus (Harper et al., 1985; De la Monte, 1988), frontal lobe (Harper and Matsumoto, 2005), and corpus callosum (Kapogiannis et al., 2012). Post-mortem analysis of rats’ brains revealed a direct relationship between the degree of WM atrophy and the amount of alcohol consumed (Yalcin et al., 2017). Chronic binge alcohol consumption impairs normal WM lipid homeostasis, such as sulfatides and phospholipids, which are crucial for maintaining myelin integrity (Yalcin et al., 2017).

Findings from pre-clinical and clinical studies suggest interconnectivity between neuroimmune response, oxidative stress, and hyperglutamatergic excitotoxicity in mediating alcohol-induced neurodegeneration. Higher levels of bacterial endotoxin lipopolysaccharides (LPS) in alcoholics induce oxidative stress by increasing reactive oxygen species (ROS) production (Crews et al., 2015; Gorky and Schwaber, 2016). In response to this, neurons and glial cells (i.e., microglia, astrocytes, and oligodendrocytes) mediate neuroimmune reactions through interactions with neuroimmune factors such as Toll-like receptors (TLRs), high-mobility group protein box 1 (HMGB1), and pro-inflammatory cytokines. Oxidative stress is further exacerbated by glutamate excitotoxicity through upregulation of glutamate receptors upon chronic alcohol intake (Kumar et al., 2016), and also damage astrocytes, which are responsible for at least 90% of glutamate reuptake in rats (Ayers-Ringler et al., 2016).

## Pathophysiology of AUD-Induced Neurodegeneration

### Alcohol-Induced Oxidative Stress in Brain

Oxidative stress is fundamental to the etiology of many diseases (Kumar et al., 2017; Liguori et al., 2018). In AUD, alcohol utilizes oxidative stress as one of the main tools to wreak havoc in central nervous system. Primarily, alcohol is metabolized by alcohol dehydrogenase (ADH) and Cytochrome P450 (CYP450) in the liver. The brain is devoid of ADH (Roberts et al., 1995), hence alcohol metabolism in brain is predominantly facilitated by CYP450 subtype 2E1 (CYP2E1) (García-Suástegui et al., 2017). CYP2E1 is localized in various cellular sites, including the plasma membrane, endoplasmic reticulum (ER), Golgi apparatus and highly expressed in the mitochondria. By-products of alcohol metabolism by CYP2E1 are acetaldehyde and reactive oxygen species (ROS), like radical superoxide anion (O${}_{2}^{-}$) and hydrogen peroxide (H_2_O_2_) (García-Suástegui et al., 2017).

Reactive oxygen species induce oxidative stress in cells and potentially damage the neurons. Endogenous antioxidants such as superoxide dismutase (SOD), glutathione peroxidase (GPX), and reduced glutathione (GSH) are important for ROS elimination. SOD inactivates radical O${}_{2}^{-}$ and forms H_2_O_2_. In the GPX reaction, GSH serves as an electron donor for H_2_O_2_ that subsequently removes ROS (Kim et al., 2015). Acute and chronic alcohol consumption decreased GSH level in the brain (Guerri and Grisolia, 1980). Prolonged oxidative stress in the brain causes neuronal dysfunction and cell death, which could lead to neurodegeneration (Bhat et al., 2015).

**FIGURE 1:**
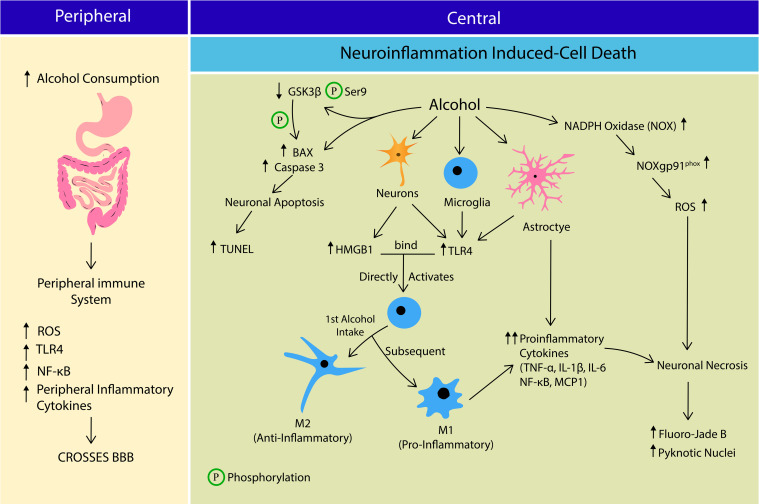
The crosstalk between peripheral and central immune system in Alcohol Use Disorder (AUD) leading to cell death. Alcohol consumption increases the peripheral production of reactive oxygen species (ROS), Toll-like receptor 4 (TLR4), nuclear factor kappa-light-chain enhancer of activated B cells (NF-κB) and inflammatory cytokines, which cross the blood brain barrier (BBB). At central nervous system, alcohol induces neuroinflammation that potentially leads to apoptosis or neurosis. Alcohol stimulates more B-cell lymphoma-2-associated X (BAX) formation, Caspase 3 expression and activates Glycogen synthase kinase-3 β (GSK3β) by reducing its Serine 9 phosphorylation. GSK3β also directly phosphorylates BAX leading to neuronal apoptosis. Alcohol stimulates neuronal release of HMGB1 and TLR4 expression on microglia, astrocytes and neurons. HMGB1-TLR4 interaction activates microglia into; M1 phenotye (anti-inflammatory) during 1st alcohol intake, and M2 phenotype (pro-inflammatory) in subsequent alcohol exposure. M2 phenotype and astrocytes releases several pro-inflammatory cytokines including tumor necrosis factor-alpha (TNF-α), interleukin 1 Beta (IL-1β), interleukin-6 (IL-6), NF-κB and monocyte chemoattractant protein-1 (MCP1). Alcohol stimulates ROS production by increasing NADPH-dependent oxidase (NOX), NOXgp91^phox^ expression and activity, leading to oxidative stress. Collectively alcohol-induced neuroinflammation and oxidative stress causes neuronal death via apoptosis and necrosis pathways.

Aside from CYP2E1, lipopolysaccharides (LPS), a serum bacterial endotoxin also was associated with alcohol consumption and oxidative stress. A previous study reported significantly high plasma LPS concentration in alcoholics who just enrolled in a detoxification program compared to control and recovering alcoholics post-2 weeks of abstinence (Leclercq et al., 2012). LPS-induced oxidative stress promotes ROS production by activating nicotinamide adenine dinucleotide phosphate (NADH) oxidase (NOX). An *in vivo* study reported an increase in NOX expression following systemic LPS administration (5 mg/kg) in mice. The LPS-induced ROS expression remained high even 20 months after the last LPS injection (Qin et al., 2013). A single high dose of LPS (5 mg/kg), given intraperitoneally (ip) shows persistent microglial activation and release of proinflammatory mediators, such as tumor necrosis factor α (TNFα), monocyte chemoattractant protein-1 (MCP-1), and interleukin-1 beta (IL-1β) in the mouse brain (Qin et al., 2013; Figure 1).

### Alcohol-Oxidative Stress-Induced Mitochondrial Apoptosis

Oxidative stress refers to an imbalance of the redox system characterized by excessive level of free radicals and impaired antioxidant system. Prolonged alcohol drinking worsens oxidative stress affecting the whole cell, or specific cellular constituents including proteins, lipids, and DNA (Hernández et al., 2016). The brain is one of the most metabolically active organs and thus, it is profoundly susceptible to oxidative stress, owing to its high demand for oxygen consumption and low levels of endogenous antioxidants to eliminate free radicals (Kim et al., 2015). Mitochondria are the major energy factory, providing at least 90% of cellular adenosine triphosphate (ATP) (Salin et al., 2015). Furthermore, the brain is abundant with phospholipids that contain a high amount of polyunsaturated fatty acids (PUFA) and therefore, is susceptible to lipid peroxidation (LPO) (Kim et al., 2015). Besides LPO, alcohol-induced oxidative stress also causes protein and DNA damage, mitochondrial dysfunction, increased cytokine production and eventually neuronal cell death (García-Suástegui et al., 2017).

**FIGURE 2:**
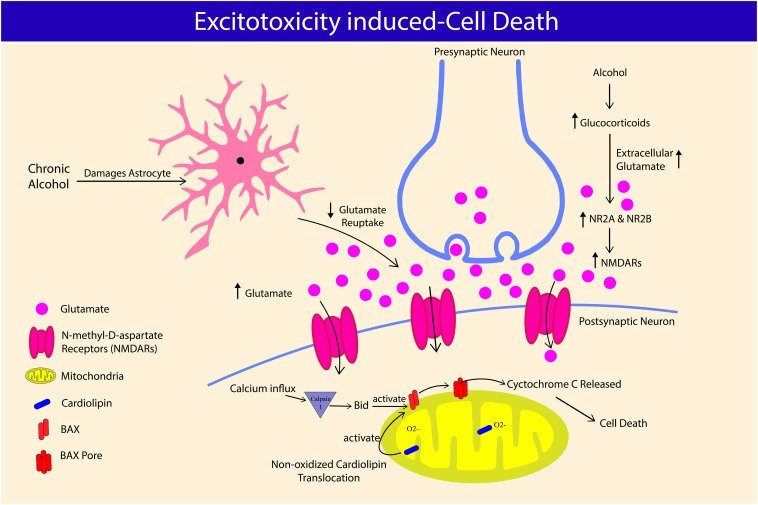
Alcohol-excitotoxicity induced cell death through glutamate and glucocorticoids. Chronic alcohol consumption damages astrocyte and its ability to regulate extracellular glutamate concentration, leading to hyperglutamatergic excitotoxicity. Chronic alcohol consumption increases glucocorticoids level, further elevating extracellular glutamate concentration. Alcohol-induced glucocorticoids also worsen alcohol-excitotoxicity induced cell death by increasing N-Methyl-D-aspartate receptors (NMDARs) and its subunit expression, NR2A and NR2B. NMDARs overactivation causes calcium influx and causes cardiolipin translocation, leading to B-cell lymphoma-2-associated X (BAX) pore formation on mitochondria, which releases cytochrome c causing neuronal cell death.

As the “powerhouse” of the cell (Salin et al., 2015), mitochondria are also the main source of ROS production (Kim et al., 2015). Oxidative stress by mitochondrial dysfunction and damage is widely accepted as one of the mechanisms that orchestrate neurodegeneration (Fischer and Maier, 2015; Figure 2). Cardiolipin (CL), a mitochondrial-specific phospholipid is believed to play a major role in the development of AUD-induced neurodegeneration. CL was found almost entirely in the inner mitochondrial membrane and necessary for maintaining normal mitochondrial functions (Xiao et al., 2017). Adult rats exposed to alcohol (20% ethanol via oral gavage for 60 days) showed significant changes in nitric oxide (NO), SOD2 and CL levels in mitochondria extracted from the brain cortex. Findings from the study revealed the nitric oxide (NO) levels to be significantly increased, while SOD2 expression reduced denoting alcohol-induced oxidative stress. Bilayer chromatography showed at least 40% decrease in CL level. The authors also reported decreased membrane-bound Na^+^/K^+^-ATPase activity suggestive of suppressed cell respiratory function. The decreased mitochondrial complex I, III, and IV activity led to mitochondrial dysfunction (Reddy et al., 2013). In the event of mitochondrial dysfunction, partial membrane potential reduction in mitochondria signals for non-oxidized CL translocation from the inner mitochondrial membrane, to the mitochondrial outer membrane (MOM) (Kagan et al., 2015). CL translocation, in return, activates B-cell lymphoma-2-associated X (BAX), which promotes cell apoptosis (Kim-Campbell et al., 2019).

### Stress-Induced Neuroimmune Response Signaling

Alcohol-induced oxidative stress initiates the innate immune response as a countermeasure by promoting the cross-talk between neurons and glial cells (i.e., microglia, and astrocytes). Prior to cell necrosis or apoptosis, affected cells release an endogenous signal namely, Danger-associated-molecular patterns (DAMPs) alarming the innate immune system for cellular damage and potential cell death (Frank et al., 2015). Chronic alcohol intake was reported to induce the neuronal release of High-mobility group box 1 (HMGB1), an endogenous DAMP (Zou and Crews, 2014). Furthermore, alcohol-induced exogenous pathogen-associated molecular patterns (PAMPs) such as LPS also trigger the innate immune system (Frank et al., 2015). In rat hippocampal-enthorhinal cortex (HEC), HDAC regulates the neuronal release of acetylated HMGB1 in response to alcohol exposure (Zou and Crews, 2014). As pattern recognition receptors, TLRs are responsible for an innate response to cellular injury and exposure to pathogens. Among various TLRs, activation of TLR4 promotes neuroimmune response and their expression on neurons, microglia, and astrocytes (Coleman and Crews, 2018). HMGB1 and LPS are ligands of TLR4 and thus stimulates the receptor, and recent studies have linked alcohol exposure and their expression on neurons. Rats’ HEC slices treated with alcohol and HMGB1 increases expression of TLR4 and inflammatory mediators such as tumor necrosis factor-alpha (TNF-α), interleukin 1 Beta (IL-1β), nuclear factor kappa-light-chain enhancer of activated B cells (NF-κB) and monocyte chemoattractant protein-1 (MCP1) (Zou and Crews, 2014), suggesting a functional link between HMGB1-TLR4 and alcohol-induced neuroinflammation in brain. Due to large molecular size, LPS is impermeable to blood-brain barrier (BBB) and therefore, thought to affect the brain via peripheral immune system (Mayfield et al., 2013; Frank et al., 2015). LPS binding to peripheral TLR4, triggers a neuroimmune response within the brain by increasing NF-κB expression and subsequent peripheral inflammatory cytokine release to cross BBB (Mayfield et al., 2013; Coleman and Crews, 2018). These findings corroborate the association between LPS and peripheral immune-to-brain signaling pathways.

HMGB1 and LPS also directly activate microglia via TLR4 recognition (Frank et al., 2015). In response to inflammatory stimuli, microglia are activated into two main phenotypes; (1) M1 or classical activation exists in an amoeboid shape and secretes pro-inflammatory factors; (2) M2 or alternate activation exists as a ramified projection and secretes anti-inflammatory factors. Activated microglia exacerbates neuroinflammatory response and promotes cell death via caspase cascade by releasing more pro-inflammatory cytokines such as TNF-α, and free radicals, suggesting a role for microglia in AUD-induced neurodegeneration (Coleman and Crews, 2018). Most studies investigating the effects of alcohol exposure on microglia activation have reported both M1 and M2 phenotypes (Ward et al., 2009; Marshall et al., 2013, 2016; Peng et al., 2017). Expression of microglia phenotypes depends on type of AUD models employed. Intermittent exposure model has reported the pro-inflammatory phenotypes, whereas the continuous intoxication model reported anti-inflammatory phenotypes (Ward et al., 2009; Marshall et al., 2016; Figure 1). A shift in microglial phenotypes was also proposed between first and subsequent binge exposure to alcohol, where first exposure results in low-grade microglial activation resembling M2 phenotype, and subsequent exposure generates more pro-inflammatory-like events such as increase in OX-42 immunoreactivity, ionized calcium-binding adapter molecule 1 (Iba-1+) cells, and TNF-α (Marshall et al., 2016). The findings suggest a more dynamic activation of microglia over time in alcohol dependent individuals with episodic binge drinking pattern. Ethanol-induced alterations in brain cytokines are also mediated by cells other than microglia, since acute minocycline injection (inhibitor of microglia) did not completely reversed ethanol-induced cytokine changes in the brain (Doremus-Fitzwater et al., 2014). In line with this finding, depletion of microglia (through administration of colony stimulating factor 1 (CSF1R) inhibitor, PLX5622) decreased expression of some neuroimmune genes (TNF-a), but not most of them (IL-1B, IL-6, Ccl2 etc.). Depletion of microglia also significantly reduced TLR2 and TLR7, but not TLR3 or TLR4 (Walter and Crews, 2017), which was thought to be mainly expressed in microglia. Apart from microglia, numerous other cells in the CNS also produce cytokines and express cytokine receptors. Inteleukin-6 (IL-6), the most consistently associated cytokine to ethanol exposure (Doremus-Fitzwater et al., 2014, 2015) is produced by numerous types of brain cells such as neurons (Schöbitz et al., 1993; Ringheim et al., 1995), astrocytes (Benveniste et al., 1990), and endothelial cells (Reyes et al., 1999). TLRs that are prominently expressed on microglia such as TLR3 and TLR4 are also expressed in other cell types, including neurons (Vetreno and Crews, 2012; June et al., 2015), astrocytes, and endothelial cells (Zhang D. et al., 2014). It is also likely that changes in secretion of cytokines depend on time (acute, intoxication, withdrawal), brain region, and blood alcohol levels. For example, TNF-α and IL-1 are significantly increased during abstinence, however, unchanged or reduced during intoxication. IL-6 was significantly expressed during intoxication, but not withdrawal. Increased expression of IL-6 during ethanol withdrawal was witnessed only in hippocampus, not in hypothalamus or cerebellum (Doremus-Fitzwater et al., 2014). Among the aforementioned cytokines, some promote pro-inflammatory response (IL-1β, TNF-α, IL-6, Ccl2) whereas some mediate anti-inflammatory reaction (IL-10, IL-4, IL-1ra, TGF-β). Therefore, it appears that CNS is placed in a continuum between pro- and anti-inflammatory events, depending on the type of cells, regions, cytokines released and stages of AUD orchestrating the neuroimmune response.

### Alcohol-Induced Hyperglutamatergic Excitotoxicity

Alcohol affects the neuronal structures and functions by directly passing through the BBB (Cornford et al., 1982; Szabo and Lippai, 2014). Alcohol alters the inhibitory actions of γ-Aminobutyric acid (GABA), and excitatory glutamatergic innervations (Kumar et al., 2013, 2016, 2018). Acute alcohol consumption suppresses and chronic intake upregulates the expression of glutamate receptors (Chandrasekar, 2013), eventually exposing neurons to injury via N-Methyl-D-aspartate receptors (NMDARs)-associated excitotoxicity (Fricker et al., 2018). Alcohol also increases extracellular glutamate concentration by limiting glutamate reuptake by glutamate transporter-1 (GLT-1). GLT-1 is accountable for at least 90% extracellular glutamate clearance (Rao et al., 2015). Post-mortem analysis of alcoholic brains shows a decreased expression of GLT-1 in the basolateral amygdala region compared to non-alcoholic brains (Kryger and Wilce, 2010). Failure to regulate glutamate intake also was implicated in neurological diseases such as Alzheimer’s disease (Maragakis and Rothstein, 2006). Overactivation of NMDARs results in a surge of Ca^2+^ influx into neurons and stimulates mitochondrial injury and death. In line with this, hippocampal slices exposed to chronic intermittent alcohol and 24 h withdrawal significantly reduced the mature neuron marker, known as Neuron-specific nuclear protein (NeuN), and nissle bodies compared to continuous alcohol exposure, demonstrating a more profound detrimental effects of excitotoxicity during the withdrawal period (Reynolds et al., 2015). Elevated intracellular Ca^2+^ activates Calpain I, a highly expressed protease in neurons. In brain, chronic alcohol intake increases calpain activity in rats’ cerebral cortex and cerebellum (Rajgopal and Vemuri, 2002), and hippocampus Dwivedi et al. (2018). Furthermore, administration of calpain inhibitor A-705253 reduced alcohol-seeking and relapse in chronic alcohol-fed rats (Vengeliene et al., 2016), suggesting the important role of caplain, not only in alcohol-induced cellular damage, but also in behavioral changes. Activated Calpain I, cleaves BH3-interacting domain death agonist (Bid) causing its activation and forming BAX pores on the mitochondrial membrane, which releases cytochrome C to cytosol and initiates apoptotic cell death via caspase cascade (Fricker et al., 2018; Figure 2).

The role of glucocorticoids (GCs) in alcohol-induced neuronal excitability and neurotoxicity also been well documented in past studies (Mulholland et al., 2005; Jacquot et al., 2008; For review, Prendergast and Mulholland, 2012). Alcohol consumption disrupts the function of hypothalamo-pituitary-adrenal (HPA) axis, which is shown through elevated blood GCs level in both humans and rodents (Errico et al., 2002; Lee et al., 2004). Circulating GCs significantly increased during ethanol withdrawal, however, little changes or mixed results were reported following chronic alcohol intake (Tabakoff et al., 1978; Adinoff et al., 2003; Little et al., 2008). In the brain, regional differences in GC level has been reported, most prominent changes noted during abstinence in hippocampus, prefrontal cortex, and thalamus across genders, and strains of rodents (Little et al., 2008). Prolonged increase in GCs was related to neuronal toxicity within the brain (Sapolsky, 2000; Erickson et al., 2003). GCs are known regulator of immune response (Sorrells and Sapolsky, 2007), was shown to reduce hippocampal dendritic complexity (Conrad et al., 2007) and cell death (MacPherson et al., 2005). GCs may cause neurotoxicity directly or indirectly via NMDARs. Chronic exposure to GCs elevates the protein expression of nucleotide-binding oligomerization domain-like receptor pyrin domain-containing 1 (NLRP-1), Caspase-1, Caspase-5, apoptosis associated speck-like protein (ASC), nuclear factor-jB (NF-jB), p-NF-jB, interleukin-1b (IL-1b), IL-18, IL-6, and microtubule associated protein 2 (MAP2) in the frontal cortex and hippocampus of male mice, suggesting GCs/NLRP-1 inflammasome-mediated neurodegeneration (Hu et al., 2016). In a separate study, GCs-mediated neurotoxicity was reported in female, but not male mice. The authors reported upregulation in GCs target genes in medial prefrontal cortex of female mice during withdrawal from chronic ethanol exposure including astrocytic genes (Wilhelm et al., 2015). Exposure to GCs increases the concentration of extracellular glutamate (Abraham et al., 2001), and expression of NMDARs subunit levels NR2A and NR2B (Weiland et al., 1997). In organotypic hippocampal slice cultures, ethanol withdrawal alone had little effects on the propidium iodide fluorescence and in cytosolic Ca^2+^ accumulation (cytotoxicity), however, exposure to corticosterone resulted in cytotoxicity regardless of the exposure period. When exposed to ethanol, or ethanol withdrawal, the cytotoxic effect of corticosterone was the greatest, suggesting the potential role of ethanol in increasing the vulnerability to corticosterone-induced toxicity. Furthermore, these effects were inhibited by RU486 (antagonist of GCs) and MK-801 (non-competitive antagonist of NMDAR), indicating the role of NMDARs in corticosterone-induced neurotoxicity in the presence of ethanol or ethanol withdrawal (Mulholland et al., 2005).

According to Collins and Neafsey (2016), alcohol-induced glutamate excitotoxicity has only been established in slice culture studies. Many investigators have attempted to understand hyperglutamatergic neurotransmission in AUD by measuring the level of glutamate or its metabolite levels, or glutamate receptor levels in human brain. Generally, brain glutamate level was reported to be low during active drinking (Ende et al., 2013; Prisciandaro et al., 2016), high during acute abstinence period (48–72 h into last alcohol intake) (Hermann et al., 2012), and abnormally low a week following the last alcohol intake (Mon et al., 2012). On the other hand, expression of glutamate receptors was found to be downregulated (Jin et al., 2014) and moderately increased (Freund and Anderson, 1996) in deceased alcoholic patients, and increased during 25 days of abstinence (Akkus et al., 2018). The closest finding to alcohol-induced neurotoxicity came from Frischknecht et al. (2017), associated significant increase in brain glutamate markers with lower gray matter hippocampal volume in both humans and rodents during abstinence. The authors associated high glutamine with reduced hippocampal volume 2 weeks after the abstinence, not during the early withdrawal phase, suggesting that neural damage could be a consequence of withdrawal, rather than intoxication. This finding is in agreement with a previous report that correlated alcohol withdrawal severity with reduced hippocampal volume during abstinence (Barnes et al., 2010). Some studies reported that brain glutamate levels normalize within longer periods of abstinence (Hermann et al., 2012; Mon et al., 2012), corresponding with improvement in brain gray and white matter volume during long-term sobriety (Pfefferbaum et al., 1995; O’Neill et al., 2001). Nevertheless, the direct link between alcohol-induced hyperglutamatergic state and excitotoxicity is yet to be proven in humans, and alcohol could negatively affect neuronal integrity through other mechanisms such as neuroinflammation and mitochondrial damage.

### Alcohol-Induced Neuronal Apoptosis

Many studies have reported significant brain shrinkage, loss of hippocampal volume (Sullivan et al., 1995; Chen et al., 2012; Ozsoy et al., 2013), and white matter atrophy in alcohol-dependent patients (De la Monte, 1988). Glycogen synthase kinase-3 β (GSK3β), a serine/threonine kinase is integral in regulation of neuronal survival and neurogenesis (Luo, 2009, 2010) and also a mediator of alcohol-induced neuronal apoptosis in the developing brain (Luo, 2012). Overexpression of GSK3β causes neuronal apoptosis in mice hippocampus with hyperphosphorylated Tau (Lucas et al., 2001). GSK3β activity is inhibited by its phosphorylation at Serine 9 (Ser9), and activated by phosphorylation at Tyrosine 216 (Tyr216) (Grimes and Jope, 2001). A recent *in vivo* study showed GSK3β mediates alcohol-induced hippocampal neurodegeneration (Ji et al., 2018). In this study, control rats expressed higher phosphorylated Ser9, whereas binge alcohol-treated rats showed dramatic reduction in Ser9 phosphorylation of GSK3β along significant decrease in number of mature neurons (NeuN) and newly formed neurons (doublecortin-positive cells) in hippocampus (Ji et al., 2018). Caspase 3 and BAX (proapoptotic proteins) are linked to alcohol-induced neurodegeneration via GSK3β. Alcohol exposure increases the expression Caspase 3 and active BAX in cerebral cortex, whereas treatment with lithium, a GSK3β inhibitor reduced both alcohol-driven Caspase 3 and Bax upregulation (Liu et al., 2009). GSK3β directly phosphorylates Bax and promotes its apoptotic action (Linseman et al., 2004). Moreover, BAX is also a known downstream target of alcohol-induced GSK3β-mediated neurodegeneration (Liu et al., 2009).

In addition to alcohol-induced apoptotic cell death, other forms of cell death such as necrosis (Obernier et al., 2002; Morris et al., 2010; Maynard et al., 2018) and even necroptosis (non-neuronal cells) (Afonso et al., 2015; Lu et al., 2016) been reported. Obernier et al. (2002) reported significant increase in markers of Fluoro-Jade B (FJB) and pyknotic nuclei (markers of cell necrosis) in all regions of corticolimbic circuit. Necrotic neurons were still apparent even two, three, and four days after the last alcohol treatment, whereas no signs of TUNEL positive cells (marker of apoptosis) were reported at any time points. Similar to these findings, Maynard et al. (2018) also reported significant increase in markers of FJB in binged male and female hippocampi, but not TUNEL, eight hours after the last intake of alcohol. Kelso et al. (2011) also reported significant increase in FJB markers in CA3 and dentate gyrus of hippocampus at two, four, and seven days after the last binge alcohol intake, and expression of FJB retuned to baseline only 14 days post binge intake. Apart from these findings, Morris et al. (2010), reported both FJB and TUNEL markers in the rodent hippocampus collected immediately after the last alcohol intake. However, the number of FJB, and pyknotic markers were higher than TUNEL, hence the authors concluded that binge-alcohol induced cell death is most likely due to necrosis, not apoptosis. In line with these findings, chronic alcohol was shown to increase microglial and astrocytes’ activation, proinflammatory cytokines (TNFa, IL-1b, IL-6) and chemokines (MCP-1), and expression of a NADPH-dependent oxidase (NOX), resulting in generation of ROS. Subsequently, activation of NF-kβ-mediated transcription exacerbates proinflammatory factors, further accentuating NOX-ROS (NOX-gp91^phox^) and NF-kβ signaling cascade, causing cell death (activated caspase-3 and FJB as markers). Administration of diphenyleneidonium (DPI; NOX inhibitor) reduced caspase-3 immunoreactivity, and FJB expression, indicating the important role of NOX-FOS in alcohol-mediated cell death (Qin et al., 2008; Qin and Crews, 2012). Based on existing literature, both apoptosis and necrosis were reported during acute, chronic binge alcohol exposure, and withdrawal across various age, and strains of rodents. Some studies reported mediators in cell death pathways (such as apoptosis), however, the ratio to necrosis or apoptosis induced cell death were not reported. Therefore, it is still unclear whether the alcohol-induced cell death is driven by necrosis or apoptosis pathway. However, considering the time, gender, and regional differences in alcohol-induced cellular pathways, the possibility of a spectrum between necrosis and apoptosis in alcohol-induced neurotoxicity should be further explored.

## AUD-Induced Neurodegenerative Disease

Motor impairments and lapses in memory are seen during intoxication and some cases of abstinence due to alcohol-induced neurological deficits (Lafortune et al., 2014). Long-term excessive intake of alcohol also attributes to more morbid neurological conditions such as Alzheimer’s disease (AD) and Parkinson’s disease (PD). Based on a Swedish National Cohort Study (1978-2008), out of 276,527 patients diagnosed with AUD and followed up for 37 years, 1,083 (0.4%) were admitted with Parkinson’s Disease (Eriksson et al., 2013). In another cohort study, conducted between 2008 and 2013 in France, AUD was the strongest modifiable risk factor for the dementia onset, encompassing 38.9% of 57353 diagnosed with early-onset dementia (Schwarzinger et al., 2018).

### Dementia or Alzheimer’s Like Phenotypes

Alzheimer’s disease is mainly a collection of proteostasis disruption, namely; Tau tangles and Amyloid-β (aβ). The formation of neurofibrillary tangles results from the aggregation of hyperphosphorylated tau protein (Boland et al., 2018). On the other hand, aβ is generated through cleavage of amyloid precursor protein (APP) by beta-secretase (BACE1) (Huang et al., 2018). In humans, studies associating alcohol consumption and development of AD have yielded mixed results. Some have associated alcohol consumption with increased risk of AD (Fratiglioni et al., 1993; Harwood et al., 1999, 2010) (For review, Piazza-Gardner et al., 2013; Huang et al., 2016; Venkataraman et al., 2017) and faster rate of cognitive decline in AD patients (Heymann et al., 2016). Some found no association between alcohol consumption and AD (Tyas et al., 2000; Ruitenberg et al., 2002; Luchsinger et al., 2004), whereas some even reported alcohol consumption as a protective factor against AD (García et al., 2010; Weyerer et al., 2011; Kim et al., 2020). At present, association between AD and AUD, is only established at pre-clinical level. Adult male Sprague Dawley rats administered with intragastric alcohol (4mg/ml) for 30 days recorded spatial reference memory and memory consolidation deficits along decrease in activities of PP2A, SOD, and increase in activity of GSK3β (hyperphosphorylates Tau), and levels of MDA, and hyperphosphorylated tau (Ser 199, Ser 396, Ser 404) in both hippocampus and cortex (Fang, 2017). Cyclin-dependent kinase 5 (Cdk5), another kinase involved in hyperphosphorylation of Tau was reported to be upregulated upon chronic alcohol exposure (Rajgopal and Vemuri, 2001), in a region-specific manner (Camp et al., 2006). Alcohol-induced increase in Cdk5 may due to downregulation of its regulators p35 or p67 (Rajgopal and Vemuri, 2001), increase in calpain activities (Rajgopal and Vemuri, 2002), which stimulates p25 (activator of Cdk5) (Kusakawa et al., 2000). Parallel to these findings, another study reported increase in BACE1 and APP expression in rats’ hippocampus following five weeks of alcohol liquid diet. Findings from the study suggest that alcohol enhances oxidative stress-induced BACE1 activity, and thus AD pathogenesis. Western blot analysis showed increased expression of proteins involved in BACE1 oxidative regulation such as presenilin (PS1) and nicastrin (Kim et al., 2011). Adult 3xTg-AD mice (humanized mice that express human MAPT, APP, and PSEN-1 transgenes and manifest AD-like pathologies) given two-bottle home-cage alcohol/saccharin (25% w/v + saccharin 0.1%, w/v) for four months showed impaired spatial memory, exacerbated conditioned fear, and diminished sensory gating in a chronic non-dependent drinking model. Laboratory findings revealed an increase in Aβ 42/40 ratio in lateral entorhinal and prefrontal cortex, total Tau expression in medial prefrontal cortex, lateral entorhinal cortex, amygdala, and phosphorylated tau expression (Ser 199/Ser 202) in hippocampus. The AD-like pathologies also associated with reduced phosphorylation of phosphoproteins associated with Akt/mTOR signaling pathway in a brain region-specific manner [amygdala: ERK 1/2 (Thr 185/Tyr 187); Lateral hippocampus: IRS1 (Ser 636), p7056K (Thr 389/412); CA1: mTOR (Ser 2448), PTEN (Ser 380); lateral entorhinal cortex: IGF1R (Tyr 1135/1736), IR (Tyr 1162/1163), PTEN (Ser 380); Medial entorhinal cortex: GSK 3α (Ser 21), IGF1R (Tyr 1135/1136), IRS1 (Ser 636), RP 56 (Ser 235/236)] (Hoffman et al., 2019). In agreement with these findings, the Akt/mTOR pathway was reported to respond selectively in a brain region-specific manner in a binge-intake alcohol dependent AUD model (Laguesse et al., 2017). Numerous other studies also have explored the function of mTOR kinases, including in mTORC1 and mTORC2 in alcohol-dependent AUD models (Beckley et al., 2016; Laguesse et al., 2017, 2018; Morisot et al., 2018; For review, Hanim et al., 2020). Future studies should extend the work of Hoffman et al. (2019) in alcohol-dependent models as factors such as blood alcohol level, animal strain, and even brain regions could greatly influence the changes in Akt/mTOR signaling pathway.

**FIGURE 3:**
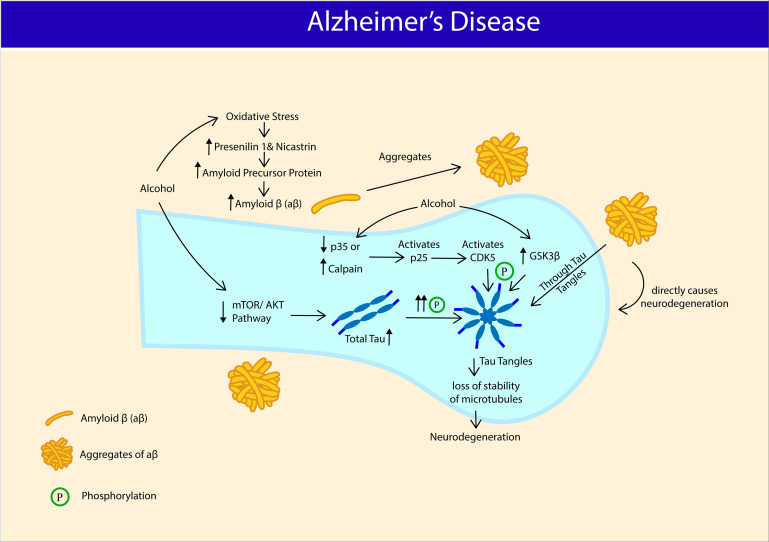
Potential association between Alcohol Use Disorder and manifestation of Alzheimer’s disease (AD). Chronic alcohol intake triggers oxidative stress, increases amyloid precursor protein (APP), presenilin (PS1) and nicastrin expression that serves as a basis for amyloid β (aβ) production and aβ aggregates. Chronic alcohol intake reduces phosphorylation of proteins associated with mTOR/AKT pathway and increases total tau expression. Chronic alcohol activates CDK5 and Glycogen synthase kinase-3 β (GSK3β) and hyperphosphorylates Tau protein into Tau tangles. Aggregates of aβ directly causes neurodenegeration and also through tau tangles. Hyperphosphorylation of tau proteins results in loss of microtubule stability, causing neurodegeneration.

Expression of TLR7, HMGB1, and microglia activation marker (CD11b) are increased in post-mortem human alcoholic hippocampal tissue and expression of TLR7 was correlated with alcohol intake. Consistent with human findings, TLR7, HMGB1, IL-1β, TNF-α, and let-b are also highly expressed in rat HEC brain slice culture following alcohol intake. Alcohol increased the release of let-7b (endogenous ligand for TLR7) in microglia-derived microvesicles and binding of let-7b to the chaperone HMGB1 and DAMP, and reduced the binding of let-7b to its classical target, Ago2. Together, the findings suggest that alcohol may mediate hippocampal neurodegeneration via let-7b/HMGB1/TLR7-associated signaling pathways (Coleman et al., 2017). MicroRNA let-7b is highly expressed in CSF of AD patients (Derkow et al., 2018). Intrathecal injection of CSF from AD patients (containing highly expressed let-7b) into the CSF of wild-type mice resulted in neurodegeneration, whereas injection into CSF of mice lacking TLR7 did not result in neurodegeneration, suggesting the pivotal role of microRNAs such as let-7b in TLR7 signaling mediated CNS damage (Lehmann et al., 2012; Figure 3).

### Parkinson’s Disease

Low to moderate consumption of beer is associated with lower risk of PD (Liu et al., 2013; Zhang D. et al., 2014), and higher liquor consumption was correlated with higher PD risk (Liu et al., 2013). However, the inverse association between low to moderate consumption of beer and lower risk of PD was only seen in males, but not females (Zhang D. et al., 2014). The protective effects of beer on PD-associated risk could be due to elevated serum uric acid level as PD was inversely associated with uric acid level, in males (Weisskopf et al., 2007). While on the contrary, higher content of pure ethanol in liquor (Cao and Prior, 2000), could act as a pro-oxidant and induce pro-inflammatory events (Mann and Folts, 2004; Liu et al., 2013). A more recent meta-analysis reported methodological weaknesses in the past studies associating alcohol intake and PD (16 articles) such as lack of statistical power, residual confounding factor, selection and recall bias, and therefore reported weak association between alcohol consumption and PD in those studies (Bettiol et al., 2015).

**FIGURE 4:**
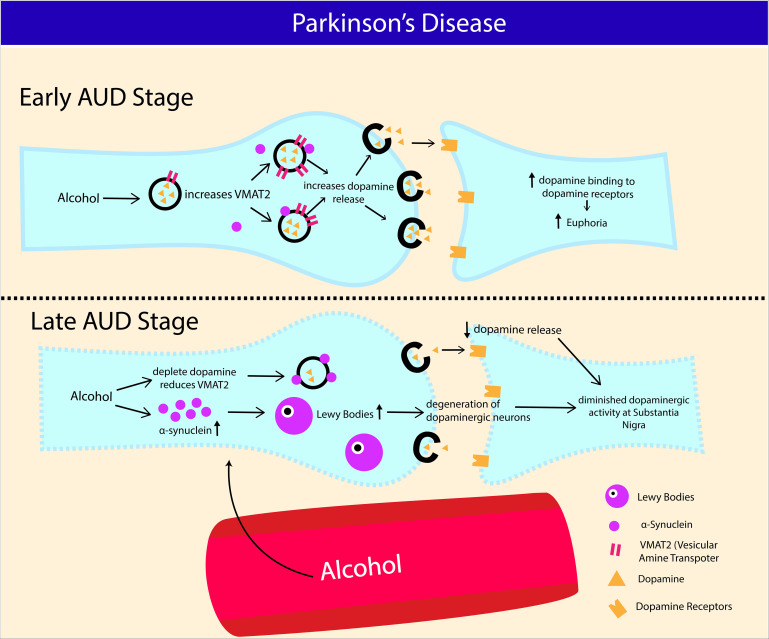
Potential association between Alcohol Use Disorder and development of Parkinson’s disease (PD). Increase in α-synuclein production and nigrostriatal dopaminergic neurodegeneration contribute to Parkinson’s disease (PD) progression. At early stage of AUD, alcohol consumption transiently increases vesicular monoamine transporter (VMAT2) activity and dopamine release in nigrostriatal reward pathway. Chronic binge alcohol intake exhausts dopamine content and consequently reducing VMAT2 activity in striatal neurons (caudate putamen and nucleus). Increased expression of α-synuclein in AUD also restricts the release of dopaminergic synaptic vesicles via reduced (VMAT2) activity. Chronic alcohol intake causes α-synuclein aggregation, leading to formation of Lewy bodies, which eventually results in degeneration of dopaminergic neurons at substantia niagra.

Apart from epidemiological associations, genetic approach also attempted to relate alcohol intake with PD. Variants in genes encoding for alcohol dehydrogenease, such as ADH1B rs1229984T was associated with increased risk of PD in women (García-Martín et al., 2019), and similar association was made with ADH4 (Buervenich et al., 2000), and ADH1C (Buervenich et al., 2005). Conversely, others found no such associations (Tan et al., 2001; Kim et al., 2020). α-Synuclein is a presynaptic neuronal protein that causes seeding of aggregation on neighboring cells contributing to progression of PD, also highly expressed in AUD patients (14.33 ng/ml; SD, 13.01 ng/ml) compared to healthy control (5.92 ng/ml; SD, 9.72 ng/ml) (Bönsch et al., 2005). In line with this, correlation between variation in SCNA gene (encoding for α-Synuclein) and alcohol craving was made, but not alcohol dependence, suggesting that the variation in the gene is not universal among heavy drinkers (Foroud et al., 2007). Some studies found no correlation between SCNA variations with alcohol dependence (Clarimon et al., 2007; Brighina et al., 2009). Although the exact role of α-synuclein in PD pathogenesis is not well delineated, prior studies demonstrated that it may restrict mobility of synaptic vesicles, hence disrupting the release of neurotransmitters via vesicular monoamine transporter (VMAT2) mediated activity (Nemani et al., 2010; Scott et al., 2010; Diao et al., 2013). The hallmark feature of PD pathophysiology is depletion of dopamine secreting neurons in the substantia nigra pars compacta of the basal ganglia and the presence of Lewy bodies (aggregated form of α-synuclein) (Davie, 2008). Dopaminergic neuron from substantia nigra pars compacta is one of the components of mesocorticolimbic system that facilitates reinforcing effects of many drugs, including alcohol (Arias-Carrión et al., 2010). Even though alcohol increases dopamine release at early stage of AUD, chronic drinking is detrimental to nigrostriatal dopaminergic neurons (Gilman et al., 1998; Eriksson et al., 2013). In line with this, VMAT2 was shown to be transiently increased during the early drug response (Boileau et al., 2008), and decreased in striatal regions such as the caudate nucleus, and putamen following chronic alcohol intake (Gilman et al., 1998), indicating lasting damage to striatal neuronal terminals in AUD. These findings were further corroborated by a preclinical work where Parkinson rats (induced by intraperitoneal injection of 1-Methyl-4-phenyl-1,2,3,6- tetrahydropyridine, 20 mg/kg) recorded the highest depletion in dopamine concentration and highest lipid peroxidation activity following 60 days of oral intake of alcohol compared to cigarette inhalation or frequent mating (Ambhore et al., 2012; Figure 4).

### Alcohol: Could It Be Neuroprotective?

A plethora of studies have reported that alcohol consumption is detrimental to health and contributes to cognitive deficits and neurodegeneration (Crews and Nixon, 2009) and increases the risk of developing neurodegenerative disorders such as AD (Harwood et al., 2010) and PD (Liu et al., 2013). On the contrary, a growing number of contemporary findings have addressed low to moderate consumption of alcohol to have neuroprotective effects (McCarter et al., 2017) and reduces the risk associated with neurodegenerative disorders (Liu et al., 2013; Zhang D. et al., 2014). Various factors were reported to determine the neuroprotective and neurodegenerative properties of alcohol, including the types of beverage consumed (beer vs wine vs liquor), amount of alcohol intake, blood alcohol level (low vs moderate vs high), nutrient deficiency and genetic components. Alcoholic beverages with lower concentration of ethanol such as beer, when taken in low or moderate amount reduce the risk of developing AD (Mukamal et al., 2003; Kok et al., 2016) and PD (Liu et al., 2013; Zhang D. et al., 2014). Compounds such as purine, niacin, folic acid, and other phenolic compounds in beer were thought to mediate the neuroprotective effects of alcohol (Sánchez-Muniz et al., 2019). Red wine, known to have high antioxidants, when consumed at low or moderate level for eight weeks (blood alcohol level: 13.9–6.9 mM) reduced microglial activation, expression of pro-inflammatory cytokines, inflammatory mediators, post-ischemic neurotrophil infiltration in a rat focal cerebral ischemic model. However, the same study also reported that the red wine failed to produce better neuroprotective effects compared to another group of rats that was given equivalent amount of ethanol (13.5%, 1.4g/kg/day), suggesting that the neuroprotective effects of low alcohol consumption was due to amount of alcohol consumed rather than the nutritional discrepancy between the beverages (McCarter et al., 2017). Even though animal models of alcohol liquid diet indicate that nutritional deficiency has little to do with ethanol-induced neurodegeneration (Crews and Nixon, 2009), human studies report the opposite, where numerous neurological and psychiatric manifestations in alcohol dependent patients were associated with chronic malnutrition (Arts et al., 2017). At present, alcohol intoxication is one of the consistently associated factors with alcohol-induced brain damage in animal models (Crews and Nixon, 2009). Binge intake (8.3 – 9.7 g/kg/day; serum ethanol level: 250–400 mg%) (Crews et al., 2004; Tajuddin et al., 2014), chronic intermittent access to alcohol (20% v/v in tap water for 26 weeks, 5–6 g/kg intake per session) (Charlton et al., 2019) have been associated with alcohol-induced neurodegeneration. Equally, in humans, consumption of beverages with high ethanol content such as hard liquor was associated with higher rate of cognitive decline (Heymann et al., 2016) and PD risk (Liu et al., 2013). In reality, most of the alcohol dependent patients practice episodic drinking pattern with more than one type of alcoholic drinks, therefore assessment of the effects of any specific types of alcohol containing drinks on their cognitive performance or neurological features is nearly impossible. In addition, rate of alcohol metabolism also varies among the individuals and impairment in metabolism of aldehydes (one of the by-products of ethanol metabolism) due to mutation in aldehyde dehydrogenase 2 (ALDH2) was identified as a risk factor for AD and PD (Chen et al., 2019; Joshi et al., 2019). Even though the growing body of literature suggests the potential neuroprotective effects of low to moderate dose of ethanol, factors such as genetic variation in metabolism of ethanol should be further explored in its role of neuroinflammation and neurodegeneration.

## Conclusion

Excessive oxidative stress is the impetus to alcohol-induced neurodegeneration and further exacerbated by excitotoxicity and neuroimmune response. Chronic binge alcohol intake accentuates glutamatergic excitatory neurotransmission directly or through HPA-axis dysfunction, resulting in neurotoxicity, especially during acute alcohol withdrawal. However, findings show that heightened glutamatergic neurotransmission recovers when the abstinence (sobriety) period is prolonged or even downregulated during craving, which leaves us to wonder about the “seriousness” of glutamatergic excitotoxicity in alcohol-induced neurotoxicity. In contrast, findings on inflammatory markers indicate that alcohol-induced neuroinflammation could mediate neurotoxicity through necrotic and apoptotic cellular cascades. It is still not clear whether microglia is the dominant player in alcohol-induced neuroinflammation, since astrocytes, neurons, and even endothelial cells express immune receptors, nonetheless, not as widely explored as the microglia in AUD. At present, the link between AUD and AD or PD is still vague at the clinical level. Prevalence of familial AD and PD cases outnumbers the sporadic events. Therefore, future studies should look into how alcohol-induced changes at the cellular level could promote expression of pro-AD or PD genes.

## Author Contributions

HK and JK contributed to the conceptual framework, design, and draft manuscript. AU and AH searched references. HK contributed to the preparation of figure. IM, GT, AU, AH, SI, MS, RM, and JK critically revised the manuscript. All authors contributed to the article and approved the submitted version.

## Conflict of Interest

The authors declare that the research was conducted in the absence of any commercial or financial relationships that could be construed as a potential conflict of interest.
